# Simplified local infill size optimization for FDM printed PLA parts

**DOI:** 10.1038/s41598-023-33181-4

**Published:** 2023-04-12

**Authors:** Márton Tamás Birosz, Mátyás Andó

**Affiliations:** Faculty of Informatics, Institute of Computer Science, ELTE Eötvös Loránd University, Budapest, Hungary

**Keywords:** Engineering, Mechanical engineering

## Abstract

The great advantage of additive manufacturing is the fact that hollowed parts with a given infill can be created. However, the standardized commercial slicer software offers a uniform infill pattern creation solution. In engineering practice, the manufactured parts are functional, therefore the appropriate load bearing capacity is mostly mandatory. In this paper a simplified local infill size optimization method has been presented. Based on a Finite Element Analysis the local density of the pattern can be adjusted, according to the emerged local stresses. The results show that independently of the pattern type, if the scaling was applied, the mechanical resistance was improved to the same extent. In case of the worst-performing uniform pattern, 84% improvement in mechanical resistance was achieved with the optimization. In addition, an FDM printing problem has been highlighted, which must be eliminated if the proposed method is used.

## Introduction

Additive Manufacturing (AM), or as commonly referred to 3D printing is a rapidly conquering manufacturing process^1^. The basic idea, that the parts can be created additively, opens a new perspective in design to create parts that are optimized for a specific function while fulfilling the requirements the best, without having to make major compromises^2^. There are much less constraints related to the shape that can be formed, since the geometry of the tool, and the movement of the machine does not affect the production, as in the case of the subtracting technologies. During AM the part is built up layer by layer, thus even a shape with more complicated surfaces can be formed. Another frequently utilized benefit is that even hollow or semi-hollowed parts can be created. This means significant raw material savings and can reduce the production time.

The infill pattern and the percentage of the filling can be easily set in almost any slicing program. Tanveer et al.^3^ concluded that in general, the higher infill density can lead to better mechanical resistance. Birosz et al.^4^ investigated the anisotropic behaviour of the simple infill patterns, and found that the orientation of the pattern doesn’t affect the mechanical behaviour. Other than basic static loads, Ötekaya et al.^5^ found the best infill ratio and pattern for vibration-damping. Their work showed that in a functional part sometimes the lower infill can be a better choice than a solid part. Apart from the conventional infill types, by forming the inner structure of the parts, they can be better perform under special conditions. Furthermore, several previous research has investigated the lightweight structures considering different aspects and using different techniques^6–8^.

Ichihara and Ueda^9^ optimized the infill structure based in a phase vector field in a bio inspired part. By depositing the material in the direction of the load path, the parts had better relative stiffnesses. If the infill is defined as the structure of the body within its boundary surfaces, the further optimizations can be classified into two bigger groups. One is the basic topology optimization, where the material connectivity within the domain is defined, such as creating holes and airgaps inside the structure^10,11^. As a result, the parts usually have strut-based structures within the initially defined design area. In this case, the voids in the part as bigger than in the conventional infill pattern, thus it’s usually not considered as an infill, even though the aim is similar, reducing the volume and mass of components. Another group is Functionally Graded Lattice Structures (FGLS), where a unit cell is created and multiplied to fill the part volume^12–14^. The density of the unit cell can determine the density of the whole part. In addition, FGLS can be used for locally compacted filling, i.e. where the load is bigger, more stress arises, thus in that location, the local density must be higher. Both solution groups are aiming to minimize the global density while preserving the stiffness of the part. However, they are computationally quite expensive and demand experts to perform the optimization. Since most of the produced part doesn’t necessarily have to be optimized to perfection such a task would be very costly, therefore the designers sacrifice the weight and material loss and print the parts as solid bodies. However, if there would be a solution to quickly and easily adjust the basic infill pattern according to the function of the produced part, it would be a nice extension of the commercial slicer software.

In this article, a simplified method is presented, and an algorithm has been created to locally adjust the infill density of the 3D printed parts according to the emerged stress field. This abridge method can be a valuable extension of the slicing and toolpath generation process before 3D printing, and can be used parallel with the optimal build orientation selection.

## Materials and methods

For the experiments, Prusament PLA Grey (Prusa Research, Prague, Czech Republic) was used. Table 1 contains the general properties of the PLA filament. This type of thermoplastic filament is one of the most frequently used raw materials for FDM printing due to its favoring printing behavior.

**Table 1:** PLA material properties.

Material	PLA
Filament diameter	1.75 mm
Printing temp	190–220 °C
Print bed	50–60 °C
Density	1.24 g/cm^3^
Color	Grey
Tensile strength	~ 47 MPa
Tensile modulus	2100 MPa

To print the parts Prusa i3 mk3s 3D printer was used. The following parameters have been set for production: 0.4 mm nozzle diameter, 0.2 mm layer thickness, 215 °C print temperature, 60 °C build plate temperature, 100% infill ratio (the creation of different infills explained in the subsection below), and four contour lines. To create the necessary g-code Prusa Slic3r 2.5 has been used. The tensile and compression tests for the geometries were performed on a Zwick Roell Z100 Universal Testing Machine. The results were evaluated using testXpert software.

**Figure 1:**
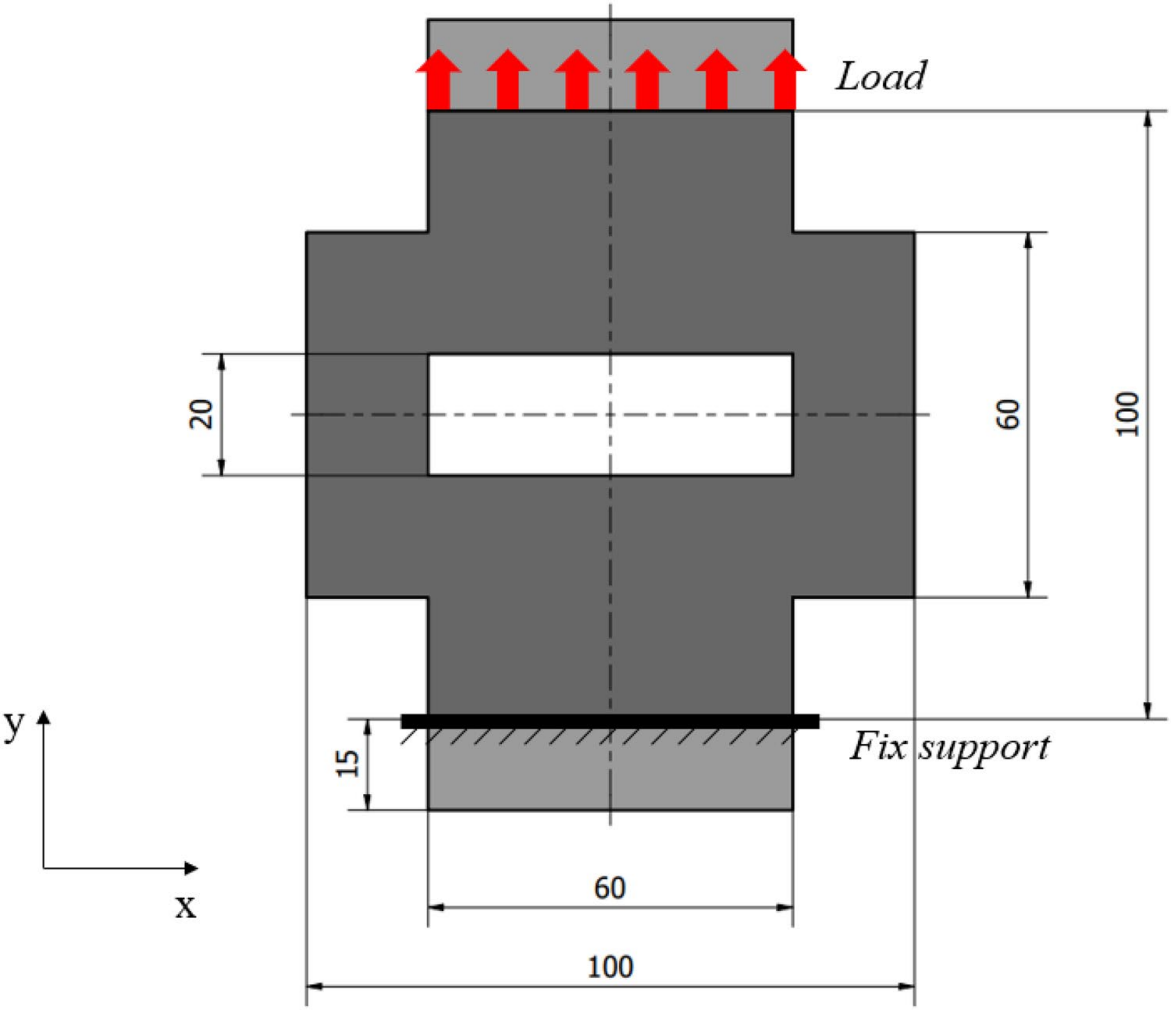
Geometry of the 2D test piece.

To test the proposed infill pattern scaling a 2D part has been used (Fig. 1), and to make it measurable 4 mm thickness has been assigned. The purpose of choosing the form was that the result of the optimization could be tested under uniaxial load on the test equipment, therefore it is symmetrical and has surfaces that can be gripped or are parallel with the contact surfaces of the grippers. Since some material has to be placed between the test machine’s grips, two extensions at the lower and top sides of the geometry have been added. These are represented in light grey color in the Figure. However, the pattern has been applied only to the dark grey regions. Since several previous research work^15–17^ investigated the anisotropic behavior of the FDM printed parts, the test geometries were printed laid on the build tray (x–y plane), so the load will act parallel to the layers, this achieving the best resistance.

### The proposed algorithm

The structure of the optimization process is the following:

1. First, a Finite Element Ansysis (FEA) has been conducted with the appropriate boundary conditions.
2. The results of the simulation were interpolated into the nodes of a predefined grid.
3. A unit cell was chosen, which composes nxn nodes. A pattern type has been selected which fills this cell and the size of the pattern in each cell has been assigned according to the emerged stresses of the composed nodes.
4. Once the locally optimized pattern size was calculated the construction of the new geometry has been executed.
5. After exporting the results, the new surface geometry has been extruded and it has been imported to the slicer software to create the necessary toolpath for the printing.

**Figure 2:**
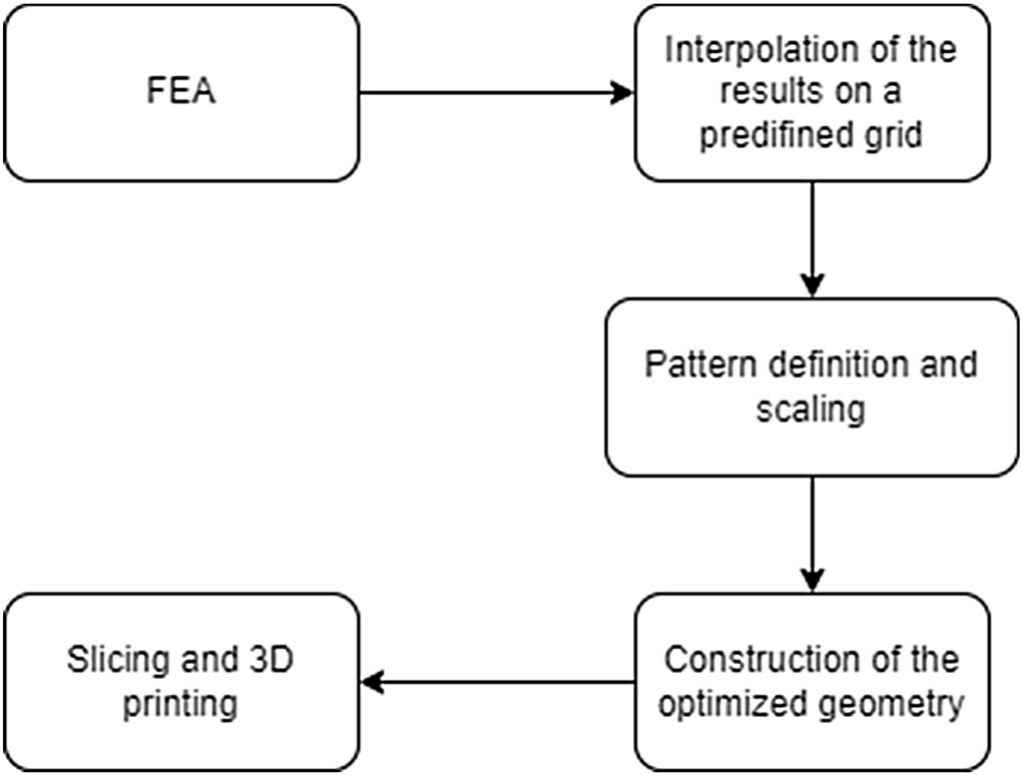
Flowchart of the proposed algorithm.

The visual representation of the process of optimization can be seen in Fig. 2.

#### Detailed description of the process steps

Since the thickness of the investigated part is relatively small compared to the other two dimensions, the FEA simulation can be done on a 2D surface geometry (plane stress). For the evaluation the tensile test machine would apply a tension load on the part, thus fixed support on the bottom edge and a force pointing in a positive Y direction was applied to the top edge. The magnitude of the force is not significant, since for the optimization only the proportional difference in different regions is necessary. For the simulation MATLAB R2022a software with Partial Differential Equation (PDE) Toolbox has been used. Since the subsequent parts of the optimization have also been done in MATLAB, it made it easy to work without manual export–import procedures.

**Figure 3:**
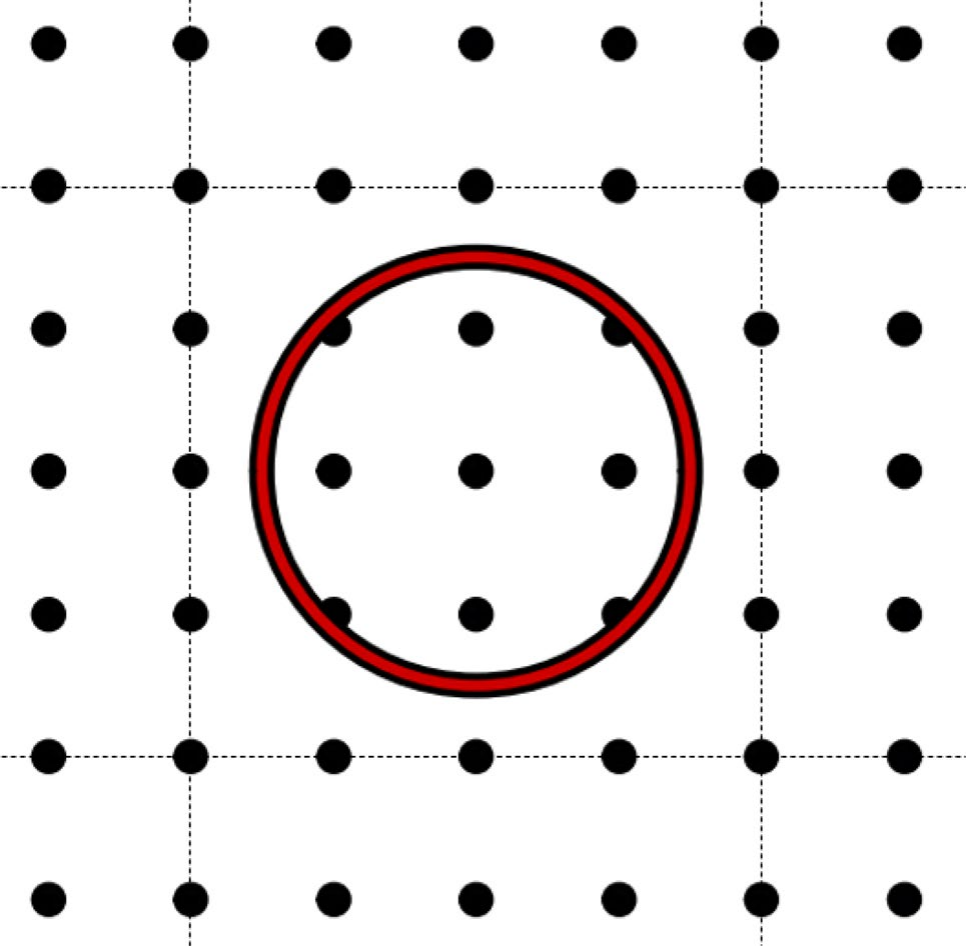
Representation of a 5 × 5 unit cell and the circular pattern.

Since the biggest dimensions of the investigated part were 100 mm, the results have been interpolated on a matrix, where the size of the unit cells is 1 mm × 1 mm. The stress values calculated on the element of the matrix have been used to appropriately assign the local infill density. First, the matrix has been distributed into equal size unit cells composed of 4 × 4, 5 × 5, and 10 × 10 nodes, which means the sizes of the unit cells were 16 mm^2^, 25 mm^2^, and 100 mm^2^ respectively for the three investigated cases. For the test, a simple circle pattern has been used, which means that in the middle of the cells a circular cut-out has been placed. Therefore, during the optimization, the location of the center point and the radius of each circle must be calculated, as can be seen in the Fig. 3.

The location of the center point is determined by the mean location of the unit cell’s nodes. A proportionality was used to determine the radius of the circles, taking into account the overall stresses in the part. Equation (1) was used to determine the significance of the cell inside the geometry and to get the corresponding radius for the local pattern,

1 $${R}_{new}={R}_{initial}(1-\frac{{\sigma }_{mean}}{{\sigma }_{max}})$$

where *R*_*new*_ is the radius of the optimized radius of the unit cell, *R*_*initial*_ is the radius that was used to start the optimization, *σ*_*mean*_ is the mean stress value calculated from the stresses of the nodes in the unit cell, and *σ*_*max*_ is the maximum stress value in the part. In this case, if relatively high stresses, close to ${\sigma }_{max}$, arise at the nodes of a unit cell (based on FEA), it means that the locations are important from a load bearing point of view. When, according to Eq. (1), R_new_ will be a number close to zero, i.e. the size of the pattern (circle) is minimal, so the loaded cross-section will be larger due to locally more material. On the other hand, if the stresses arising in the nodes that make up a given unit cell are negligible, then in Eq. (1) R_new_ is almost the same as the starting radius (R_initial_), that is, the size of the sample circle is also maximal. The value for *R*_*initial*_ has to be chosen to get the desired infill density at the end of the optimization. Since its determination is not necessarily, before the start of the optimization process, at this point the algorithm steps into an iterative phase. If the sum area of the circles is bigger than 1-(Infill ratio*Solid part area), the value of *R*_*initial*_ must be lowered, and vice-versa. The iteration continues until the optimized pattern with *R*_*initial*_ gets a density, which is the pre-set Infill ratio (± 0.1). For the test 75% infill ratio was chosen, so the overall area of the circles should be 25% of the surface geometry’s area.

Once the radius and location of the centers of each circle are defined, the circles must be extracted from the surface geometry. The resulting perforated surface is extruded to 4 mm and saved into an .stl file, which was imported to the slicing software and the toolpath with the given printing parameters is created. Furthermore, for the comparison bases, for each three pattern sizes, a uniform pattern version has been created.

## Results and discussion

**Figure 4:**
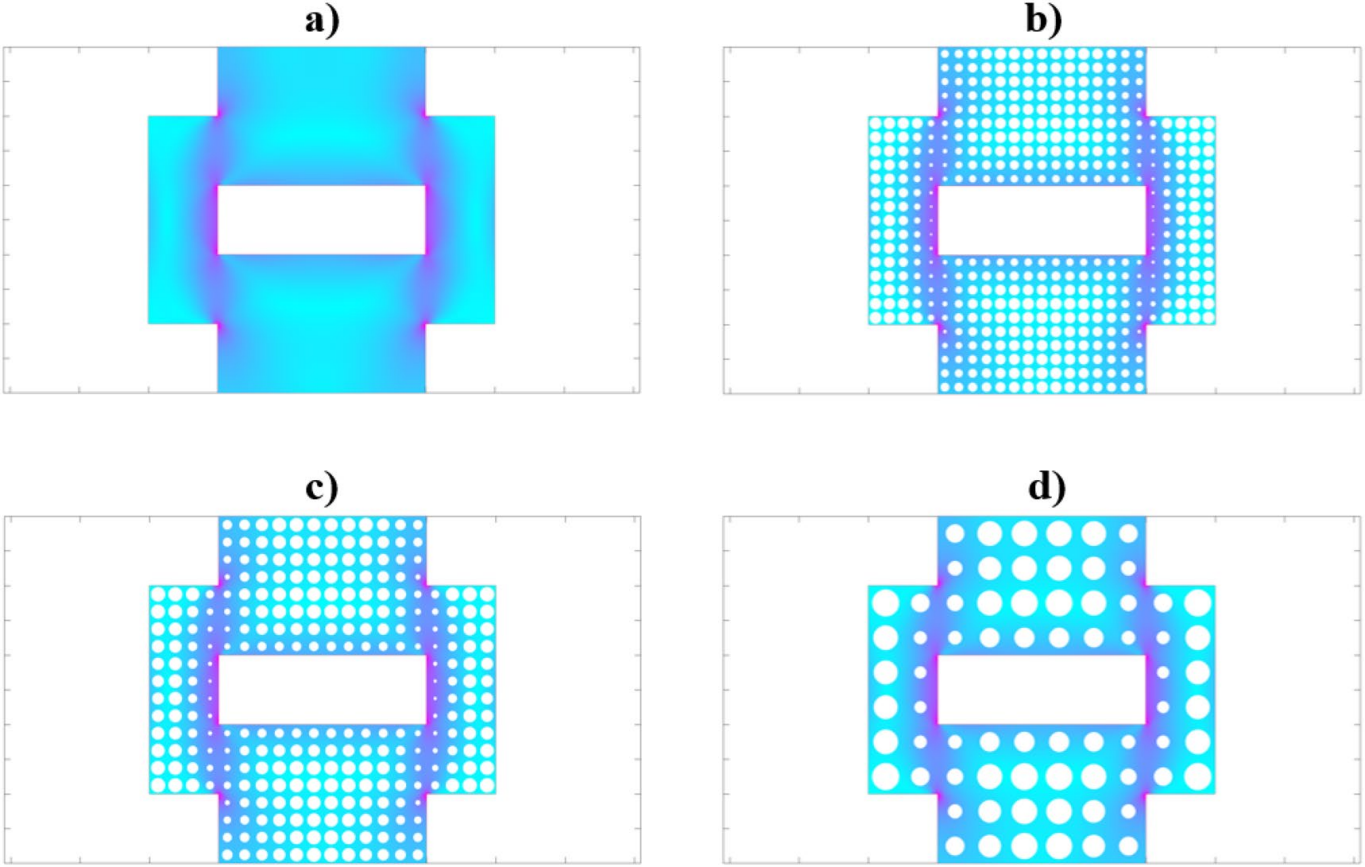
Results of the optimization (**a**) original surface geometry, (**b**) optimized with 4 × 4 unit cell, (**c**) optimized with 5 × 5 unit cell, and (**d**) optimized with 10 × 10 unit cell.

The FEA results of the tested shape can be seen on Fig. 4.

It can be observed that there are some stress singularities at the sharp corners of the shape. This doesn’t necessarily affect the effectiveness of the optimization. Also due to the cut-out in the middle, the simple load case resulted in a complex stress field. Some regions are more important in the geometry, they are represented with a darker colour, and with lighter colour the regions where the existence of material is almost negligible.

In the Figure above b, c, and d the optimized circle patterns have been applied to the FEA plot of the initial geometry. This representation shows that the circles are smaller where there is a bigger need for material. As it can be easily guessed, there are much fewer stresses in the regions of the external corners. They are far away from the load path, so the pattern size can be maximal. It can be observed, that if the pattern resolution is smaller, a more accurate scaling can be applied. In case, of the 10 × 10 unit cell at some locations, the cell consists of nodes with high stress and low stress at the same time. This would result in inaccurate optimization in specific cases.

**Figure 5:**
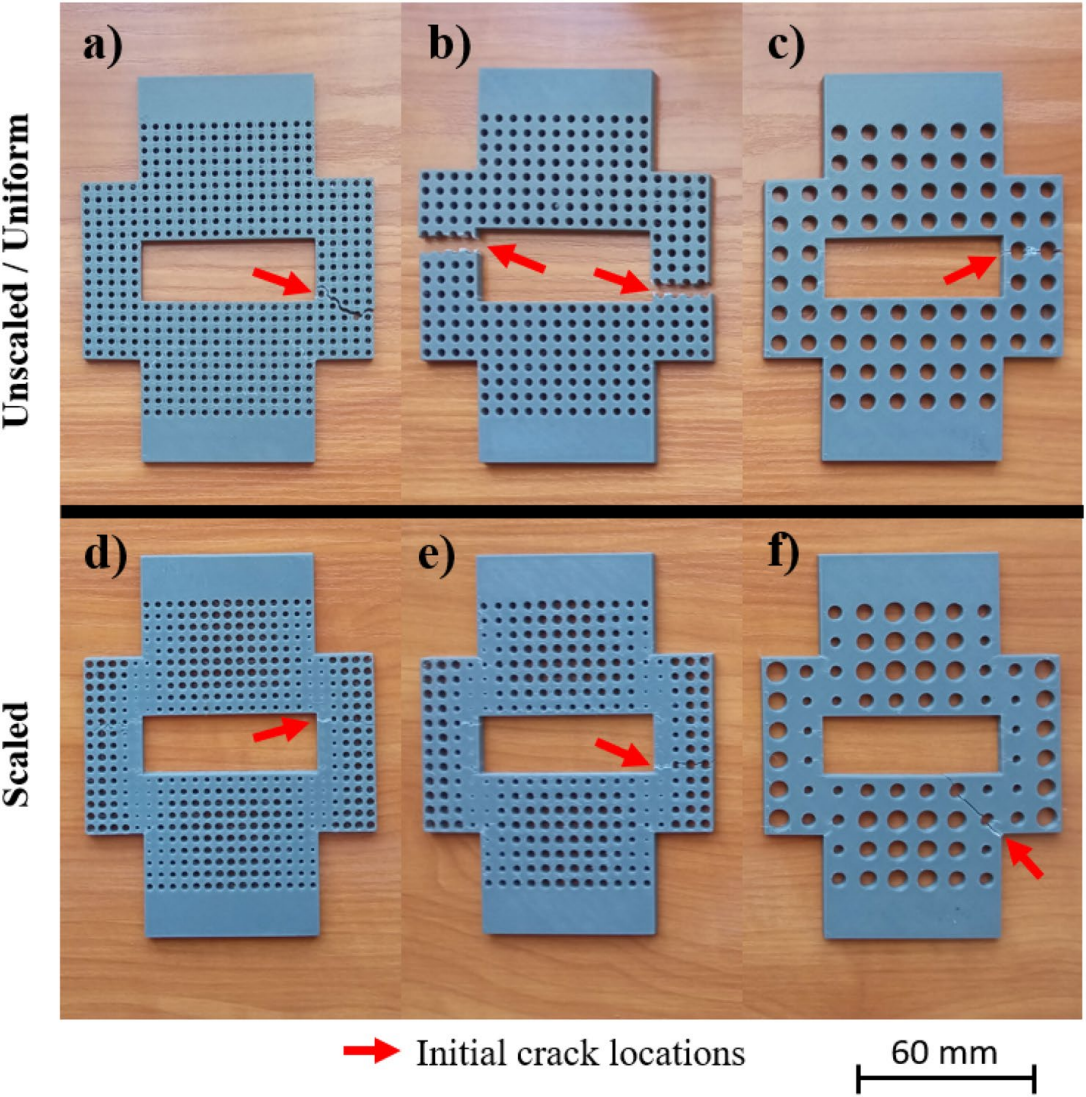
Broken test pieces (**a**) 4 × 4 unscaled, (**b**) 5 × 5 unscaled, (**c**) 10 × 10 unscaled, (**d**) 4 × 4 scaled, (**e**) 5 × 5 scaled, and (**f**) 10 × 10 scaled.

In Fig. 5 the broken test pieces can be seen. The measurement was stopped at 50% force drop, so the fracture of the parts is not complete. However, the main purpose was to determine what is the maximum load that the geometry with the applied pattern can resist. The red arrow on the figure point to the locations where the initial cracks started.

**Figure 6:**
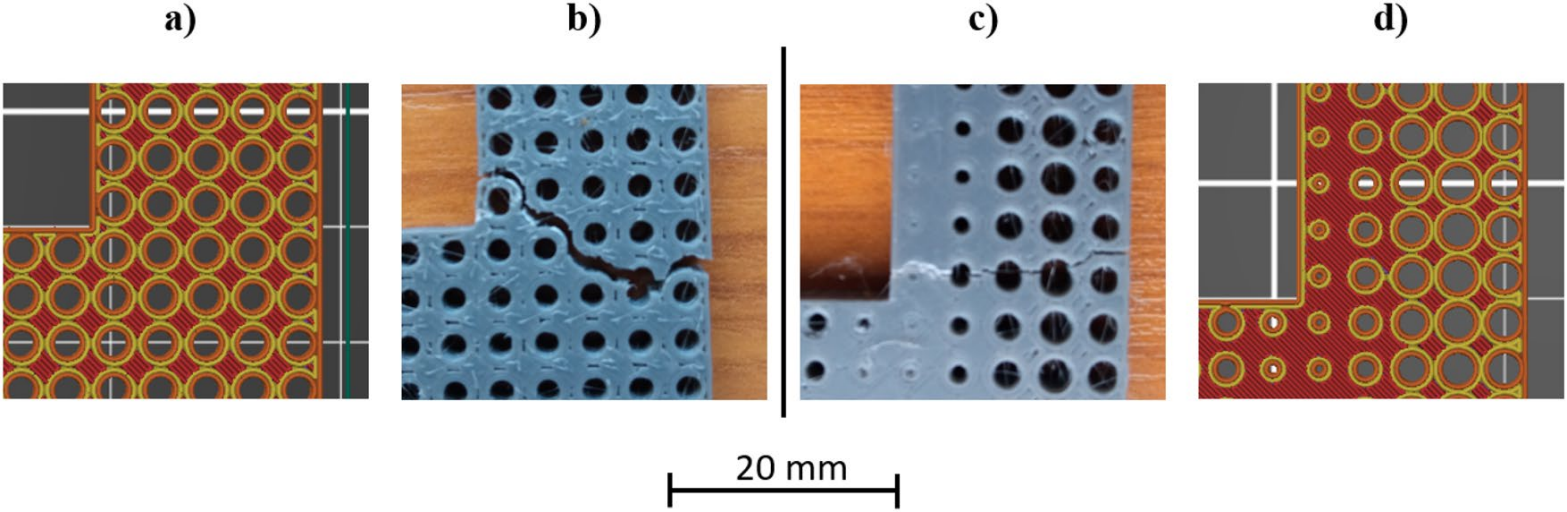
Peculiarity of FDM technology (**a**) generated toolpath for 4 × 4 unscaled specimen, (**b**) broken region of 4 × 4 unscaled specimen, (**c**) broken region of 4 × 4 scaled specimen, and (**d**) generated toolpath for 4 × 4 scaled specimen.

As was expected the locations are concentrated in the regions, where stress was higher, e.g. at the corners. In general, two different fracture types can be distinguished. The one is when fractured surfaces are mostly straight, due to the relief, the initial crack starts, and the sample breaks in one line. The second is when, regardless of the initial crack location, the fracture follows the discontinuity of the material, which is not always mean the voids due to the pattern, but a manufacturing error. The basis of this manufacturing error is shown in Fig. 6. This characteristic is most prominent in the case of the 4 × 4 scaled and unscaled samples, thus on the figure the location of the failure and the associated toolpath representation extracted from the slicer software has been presented.

As can be seen, the machine first draws the contour lines of the layer, then fills the region between the contours according to a zig-zag path. As an initial setup, two contour lines, inner and external contour, have been used to produce the test specimens. If the generated circles in the adjacent unit cells are big, a solid infill cannot be formed between them. Therefore, only the external contours are connecting with each other. Also, it can be observed that there are smaller material discontinuities near the locations where the adjacent pattern circles are tangentially connected. This is due to the fact that the print head cannot deposit material into those locations without creating serious material overlapping. This further weakens the component’s resistance, in addition to the infill pattern. It is clearly visible in Fig. 6b) that the fracture happened between the pattern’s circles, which is the result of the weak adhesive connection inside the part. Similarly, in the 4 × 4 scaled part, the initial crack happened between the unit cells, but the propagation of this crack towards the interior of the part turned into the breaking of the pattern since a solid infill was created between the unit cells. This problem could be eliminated, if the number of the contour lines is reduced to one, or a bigger infill ratio is selected. Another observation is that the FDM technology has difficulties printing small details. The optimized pattern’s circle that has a minimum radius was formed, but during printing mostly it’s like they have merged. It’s not a problem per se, however, it must be borne in mind that maybe it’s better to delete the circle from that unit cell if it’s too small, since they are not significant anyway, and some manufacturing problems can also be eliminated this way. However, the specimens with 10 × 10 unit cell are free from these harmful effects, since the sample size is sufficiently large, and the contour lines do not have such an adverse effect either.

**Figure 7:**
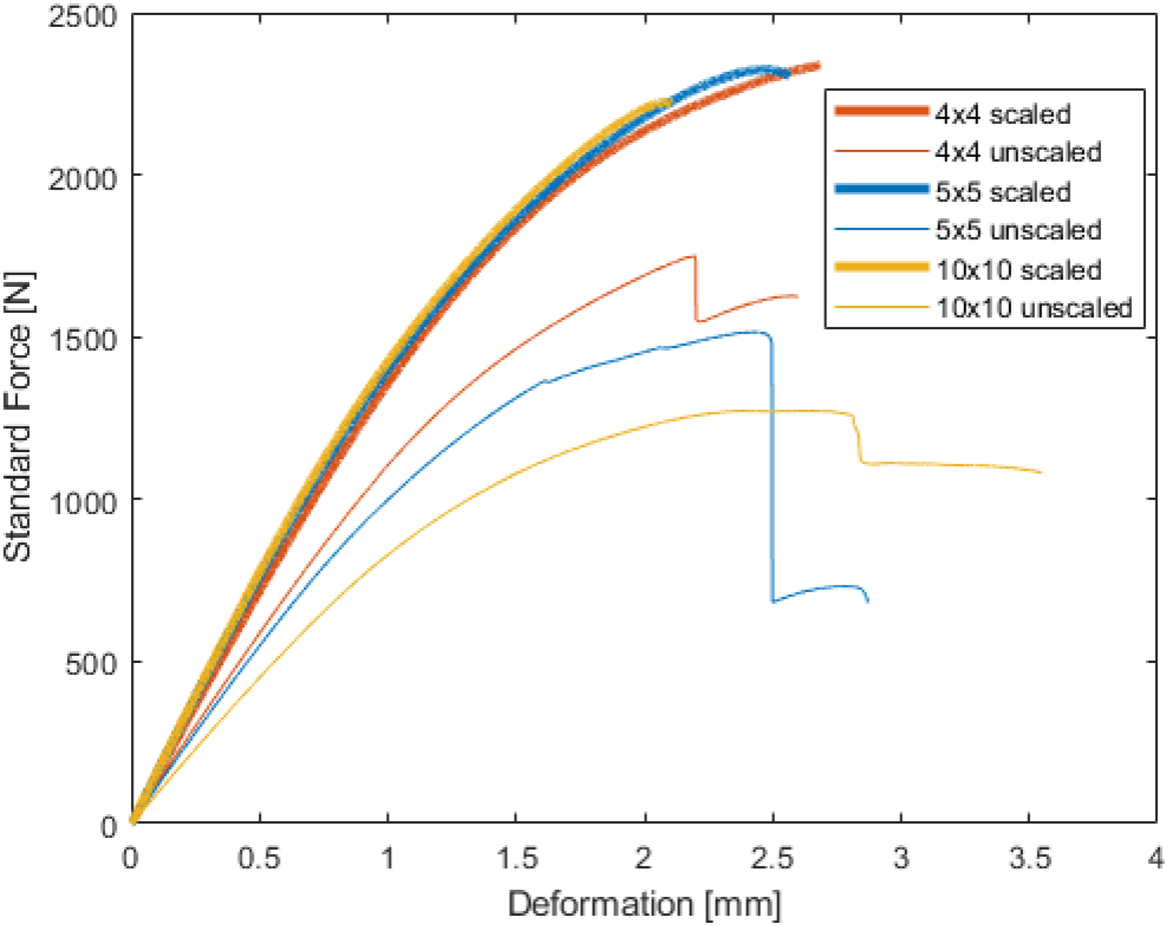
Results of the tensile test.

The results of the tensile tests can be seen on Fig. 7.

It can be seen that all of the scaled patterns have bigger rigidity. In the curves of the scaled patterns, regardless of the size of the unit cells, the mechanical resistance is almost the same. This proves the concept that based on the emerged stress field, local enhancement can significantly increase the load-bearing capacity of the parts while preserving the same infill ratio.

Also, interesting to see the effect of the unit cell’s size. In case, of the unscaled pattern, the bigger size decreases the mechanical resistance. Which background is that there is less material in the necessary areas which makes it more flexible the parts. In case, of the scaled pattern, where higher stress occurs, there is more material in any size case—there is no weakening effect and the parts become more rigid. The big advantage of this method is that the user can select the unit cell’s size according to the other requirements and remained the excellent load-bearing capacity of the workpiece. There are limitations of this method, but don’t forget, if there are narrow areas the stress becomes higher and the bigger unit cell also has only a small circle—as can be seen in the case of internal corners.

A significant increase in the production time was not experienced for any of the variations, since the number of unit cells remained the same. Thus, the printhead had to accelerate and decelerate approximately the same number of times during the formation of the layers. As a result, when choosing the size of the pattern, secondary aspects such as ergonomics, work safety, or visual appearance may be more dominant.

## Conclusion

In this paper, a simplified infill pattern optimization has been presented. Based on Finite Element Analysis, the local density of the infill pattern has been scaled, according to the emerged stress field inside the part. Based on the results the following conclusions can be drawn:

- The FDM layer formation method highly affects the breaking type of the parts. If it’s aimed that the produced components need acceptable mechanical resistance, it’s advised that the size of the infill pattern must be small enough to print solid infill between the unit cells.
- Scaled patterns give higher resistance to the workpiece without any negative effect on the outer surface or final mass.
- Once a scaling method is applied, the load resistance became independent of the pattern size, which gives high design flexibility.
- In case, of an unscaled pattern, a small size should use to not ruin more the rigidity.

## Data Availability

The datasets used and/or analysed during the current study available from the corresponding author on reasonable request.
